# Management options for interstitial ectopic pregnancies: A case series

**DOI:** 10.12669/pjms.332.12093

**Published:** 2017

**Authors:** Ilker Kahramanoglu, Zahid Mammadov, Hasan Turan, Aslihan Urer, Abdullah Tuten

**Affiliations:** 1 Ilker Kahramanoglu, MD. Department of Obstetrics and Gynecology, Cerrahpasa Medical Faculty, Istanbul University, Istanbul, Turkey; 2 Zahid Mammadov, Department of Obstetrics and Gynecology, Cerrahpasa Medical Faculty, Istanbul University, Istanbul, Turkey; 3 Hasan Turan, Department of Obstetrics and Gynecology, Cerrahpasa Medical Faculty, Istanbul University, Istanbul, Turkey; 4 Aslihan Urer, Department of Obstetrics and Gynecology, Cerrahpasa Medical Faculty, Istanbul University, Istanbul, Turkey; 5 Abdullah Tuten, Associate Professor, Department of Obstetrics and Gynecology, Cerrahpasa Medical Faculty, Istanbul University, Istanbul, Turkey

**Keywords:** Ectopic pregnancy, Interstitial pregnancy, Methotrexate

## Abstract

**Objective:**

Ectopic pregnancy in the interstitial part of the Fallopian tubes can be life-threatining considering the thin myometrial tissue surrounding the gestational sac and highly vascularization which may result in catastrophic haemorrhage when interstitium is ruptured. The diagnosis and management is challenging. Conservative, medical, and surgical treatment options should be considered based on individual patient factors.

**Methods:**

Four women were diagnosed with interstitial pregnancy in last five years in our tertiary center. Four different treatment modalities, including single dose methotrexate, laparotomy, hysteroscopy followed by vacuum aspiration, and vacuum aspiration under laparoscopy were performed according to patients’ characteristics.

**Results:**

. Successful outcome was achieved in all patients.

**Conclusion:**

Interstitial pregnancy can be successfully treated with a single dose systemic methotrexate when all criteria are met. The classical cornual wedge resection remains lifesaving operation for cases of ruptured interstitial pregnancy. Less invasive procedures such as laparoscopic assisted transcervical vacuum aspiration and diagnostic hysteroscopy followed by vacuum aspiration can be performed in selected cases.

## INTRODUCTION

Interstitial pregnancy is a rare type of ectopic pregnancy that accounts for 2-4% of all ectopic pregnancies, and almost 20% of all deaths caused by ectopic pregnancy.^1^ Interstitial pregnancy results when implantation occurs in the interstitium, the most proximal part of the fallopian tubes that is surrounded by the myometrium.^2^

Although in some American and European literature, the term ‘interstitial pregnancy’ is used as a synonym to ‘cornual pregnancy’, they should be considered as two different clinical situations; however, a pregnancy is referred to as a cornual pregnancy if the implantation occurs in one horn of a bicornuate uterus or in one-half of a septate or sub-septate uterus.^2^

Most of the risk factors for interstitial pregnancy are similar to those for all types of ectopic pregnancies in general, including previous ectopic pregnancy, pelvic inflammatory disease, and previous pelvic surgery, the use of assisted reproductive technologies, sexually transmitted diseases and ovulation induction. Ipsilateral salpingectomy is a unique risk factor for interstitial ectopic pregnancy.^1^

Considering the 2.5% mortality rate, early detection is crucial.^3^ Interstitial pregnancy must be distinguished from angular pregnancy, which occurs when the embryo is implanted medial to the uterotubal junction in the lateral angle of the uterine cavity, close to the internal ostium of the fallopian tube.^2^ A diagnosis of interstitial pregnancy can be made when the following ultrasonography signs are seen: empty uterine cavity, a gestational sac separate and at least 1 cm from the lateral edge of the uterine cavity and a thin (<5 mm) myometrial layer surrounding the chorionic sac.^3^ ‘The interstitial line sign’ that extends from the upper region of the uterine horn to border the intramural portion of the fallopian tube has also been used in the past for diagnosis of this rare type of ectopic pregnancy.^4^

There is no standard care for interstitial pregnancies. There are several treatment methods, including systemic methotrexate (MTX) treatment, direct injection of MTX into the gestational sac and combined systemic and direct injection technique for unruptured cases.^2^ Different treatment modalities have also been reported for patients desiring fertility preservation in case of systemic MTX treatment failure: laparoscopically assisted transcervical suction evacuation and hysteroscopic-guided removal of conception products. However, ruptured cases are managed as urgent cases; cornuostomy, cornual resection or hysterectomy is done to handle the life-threatening bleeding.^5^

Interstitial pregnancies present a difficult management problem with no absolute standard of care; several treatment modalities are proposed, both surgical and medical, and different cases have been reported with variable management, but the most appropriate technique for the treatment of these ectopic pregnancies remains controversial.^2^,^5^ Therefore, on the basis of the available literature, there is a need for more data to determine the best management option for interstitial ectopic pregnancy. In this case series, we describe different management options in four patients with interstitial pregnancy.

## CASE REPORTS

### Case 1

A 33-year-old woman, gravida 8 para 4 abortus 4, was admitted to our tertiary centre with complaints of vaginal spotting and mild lower abdominal cramping. She was in her third week of amenorrhea.

Her obstetric history included four caesarean sections. The patient’s medical history was unremarkable, and her vital signs were within normal limits. The abdominal and vaginal examination revealed mild lower-right quadrant tenderness, and no significant vaginal bleeding was noted. Hemoglobin, complete blood count and serum biochemistry including liver enzymes, blood urea nitrogen and creatinine levels were within normal limits.

**Fig.1:**
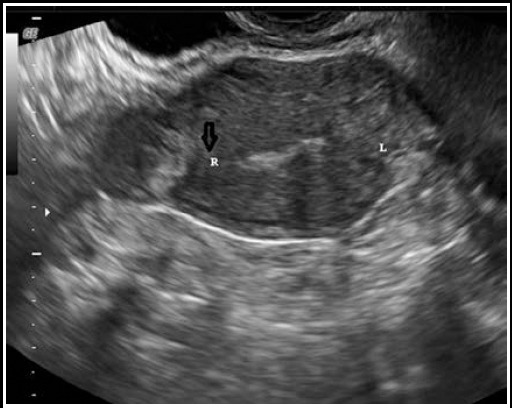
Coronal view of the fundus of the uterus on transvaginal ultrasound showed a mass measuring 2 cm in the right uterine horn and the interstitial line sign (black arrow).

At the time of admission, serum level of beta human chorionic gonadotropin (ß-hCG) was 1263 IU/L. Transvaginal ultrasound revealed an empty uterine cavity and a mass of 20×19 mm with a hypoechoic central area in the right horn (Fig.1). No sign of foetal pole or yolk sac was noted. The Doppler showed the vascular ring sign, which proved an intense peripheral vascularization. Both ovaries appeared normal. No free fluid was noted in the Pouch of Douglas.

Considering the serum level of ß-hCG (<10000 IU/L), the patient’s haemodynamic stability, the size of the mass (diameter <4 cm), the absence of pelvic free fluid and the absence of haematologic, renal and hepatic impairment, systemic MTX therapy was recommended as a first option for treatment. The patient was given an intramuscular MTX (Methotrexate; Atafarm, Istanbul, Turkey) 50 mg/m^2^ (Day 1). She was hospitalized for seven days after MTX injection and remained asymptomatic during this period. There was a more than 15% decrease in ß-hCG levels between the 4^th^ and 7^th^ days after injection (654 IU/L and 464 IUI/L on Day 4 and Day 7, respectively). In addition, transvaginal ultrasound revealed reduction of peripheral vascularization, and the ectopic mass became more echogenic with the same size. No pelvic free fluid was noted, and the patient did not have any side effects after treatment.

The ß-hCG level became negative 45 days after MTX injection. Follow-up ultrasonography at two months after treatment showed a significant reduction (9×6 mm) in the size of the mass and no peripheral vascularization around it. The mass could not be visualized at the third month after treatment.

### Case 2

A 25-year-old gravida four para two presented at our emergency department with complaints of generalized abdominal pain of six hours duration of acute onset and dizziness. The pain was aggravated upon lying down and radiated to both shoulders. She was in her second week of amenorrhea.

Her obstetric history included one pregnancy termination at 18^th^ weeks due to multiple foetal anomalies three years prior. In addition, she had undergone laparoscopic right salpingectomy due to right tubal pregnancy two years before.

On examination, a mild degree of pallor was noticed. Her pulse and blood pressure were 105 bpm and 90/60 mm Hg, respectively. Her abdominal examination revealed generalized tenderness. Her vaginal examination revealed cervical tenderness, and her fornices were full. Transvaginal US examination revealed an empty uterine cavity, free abdominal fluid and coagulum in the cul-de-sac. At the time of admission, serum level of ß-hCG was >10000 IU/L. A *complete blood count* on the *day* of *admission revealed a hemoglobin level of 6 g/dL*.

**Fig.2:**
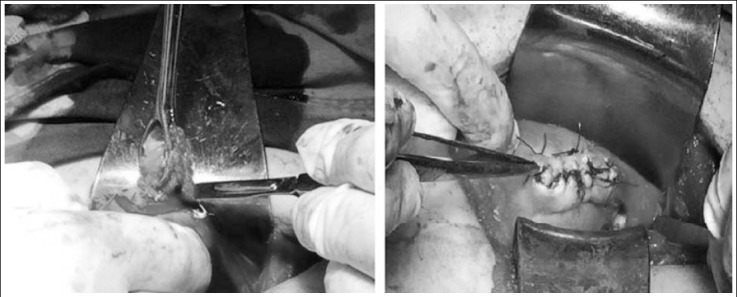
Laparotomy revealed a ruptured right uterine horn. (a) The ectopic mass was clamped and cornual resection was performed. (b) Resected segment of uterus was repaired.

The clinical and ultrasonographic findings were suggestive of ruptured ectopic pregnancy. The patient’s consent was taken for an emergency exploratory laparotomy, which showed a massive hemoperitoneum and a ruptured right interstitial pregnancy. The site of the bleeding was clamped, and cornual resection with the conception products inside was done (Fig.2). Uterine cornu was sutured separately with 0-Vicyrl. The ovaries, left fallopian tube one1 unit of fresh frozen plasma.

The postoperative period was uneventful. Her *hematological* and clinical *biochemistry values were within normal range on her immediate postoperative follow-up and postoperative day 1*. The patient was discharged on the third postoperative day and called for the control examination a week later. The control examination findings showed no significance, and the histopathology report of the specimen confirmed interstitial ectopic pregnancy. Serum β-hCG level decreased by 98 % at postoperative day 7.

### Case 3

A 39-year-old gravida 5 para 2 abortus 2 was admitted to our clinic at her eighth week of amenorrhea. Her obstetric history included two normal spontaneous deliveries, and her general condition was stable. Her complaints included vaginal bleeding, left-lower quadrant pain and 17 days of amenorrhea. Abdominal examination revealed left-lower quadrant tenderness. Bleeding from the cervical canal was noted.

**Fig.3:**
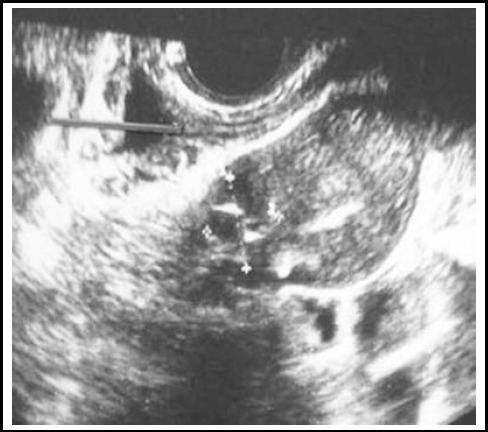
Transvaginal ultrasound image (coronal plane) showed a gestational sac in the left uterine horn.

At the time of admission, serum level of ß-hCG was 9277 IU/L. Transvaginal ultrasound showed a mass in the left uterine horn and an empty endometrial cavity (Fig.3). The yolk sac and foetal pole were also noted within the gestational sac. Crown-rump length (CRL) was measured 5.9 mm. No foetal cardiac motion was noted.

The Doppler showed significant peripheral vascularization. The ovaries appeared normal bilaterally, and no free fluid was noted in the Douglas pouch. *Serum* chemistry values and *complete blood* counts *were* within normal limits. The patient was counselled on all available management options, including minimally invasive laparoscopic, hysteroscopic and medical management. She elected first for hysteroscopic exploration and transcervical evacuation under laparoscopic guidance if needed.

After obtaining consent for all of the procedures listed above, hysteroscopy was performed with a 5-mm operative hysteroscope with a 2.9 mm 30º lens (Karl Storz, Tuttlingen, Germany) and normal saline as the endometrial distending media. No pathologic lesion was visualized within the endometrial cavity. The right tubal ostium was identified, and a coagulum-like lesion was visualized in the left tubal ostium bulging into the endometrial cavity. At that point, the procedure was terminated. The cervical canal was dilated to 8 mm, and an 8 mm flexible cannula was placed into the endometrial cavity just near the gestational sac under direct abdominal ultrasound visualization. Vacuum aspiration was performed, and the conception product was removed with several passes. Histopathologic examination results reported necrotic chorionic villi. There was no need for laparoscopy because the abdominal and vaginal ultrasonography provided sufficient visualization.

No complications were noted during the procedure, and the patient was discharged on the same day. She was monitored for serum level of ß-hCG, which was identified to be 156.3 IU/L at the tenth day, 35.01 IU/L at fifteenth and 1.68 IU/L at twenty-fifth day after the procedure.

### Case 4

**Fig.4:**
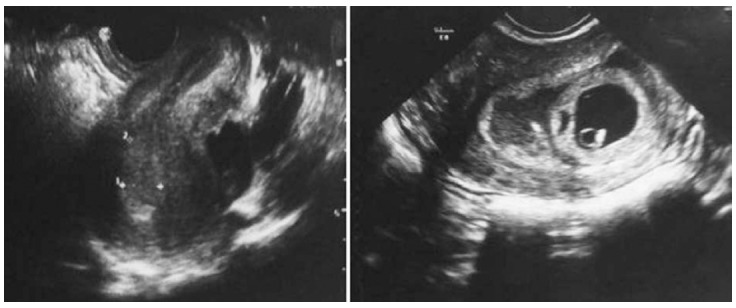
The endometrium with trilaminar pattern on sagittal axis. (a) and a left-sided interstitial pregnancy on coronal axis (b) within CRL measured 31 mm, and foetal cardiac motion was detected.

A 28-year-old gravida one para 0 was referred to our tertiary centre with a diagnosis of ectopic interstitial pregnancy. She complained of amenorrhea for seven weeks and lower abdominal pain. Her abdominal examination revealed lower abdominal tenderness bilaterally. Vaginal examination was normal, but transvaginal ultrasonography revealed left-sided interstitial pregnancy, with gestational sac and foetal pole within. CRL measured 31 mm, and foetal cardiac motion was detected (Fig.4). The myometrial thickness measured 3 mm around the gestational sac. A subchorionic haematoma with dimensions of 44×11 mm was also visualized near the gestational sac. No free abdominal fluid was noted.

The patient’s general condition was stable. Her pulse was 100 bpm, and her blood pressure was 100/70 mm Hg. *Serum* chemistry values and *complete blood* counts *were* within normal limits. Laparoscopy was performed. A 5 mm incision was made in the umbilicus, and a Veress needle was inserted to obtain pneumoperitoneum. The Veress needle was replaced with 10 mm trocar after confirmation of direct intra-abdominal placement laparoscope was made. An auxiliary 5 mm port was opened thereafter. The exploration revealed bulging of the left cornual region lateral to the round ligament. Bilateral ovaries and fallopian tubes remained normal. No gross placental accreta sign, blood or coagulum was noticed. With the findings mentioned above, we decided to perform transcervical suction under laparoscopic visualization. The cervical canal was dilated to 8 mm, and an 8 mm flexible cannula was placed into the endometrial cavity. The products of conception evacuated with three passes. Frozen pathology revealed products of conception. The left cornual region remained unruptured during the procedure. The patient tolerated the operation well and was discharged the next day. The serum level of ß-hCG was detected as < 5 IU/L on the seventh day after the procedure.

### Informed consent

Informed consent was obtained from all of the patients involved in this case series.

## DISCUSSION

Summaries of the four cases with interstitial pregnancy that were treated at Istanbul University Cerrahpasa Medical Faculty, Department of Obstetrics and Gynecology between 2012 to 2016 are presented in Table-I. All of the patients were counselled on all available management options including expectant, medical and surgical management.

**Table-I:** Summary of patients with interstitial pregnancy.

	*Age (years)s*	*Predisposing factor*	*Presenting symptom*	*Initial serum levels of ß-hCG (IU/L)*	*tvUS*	*Treatment*	*Outcome*
Case 1	33	None	Vaginal spotting, mild lower abdominal cramping and third week of amenorrhea	1263	A mass of 20×19 mm with a hypoechoic central area in the right horn with a peripheral vascularization	Single dose MTX	Successful Negative ß-hCG levels at 45 days after MTX
Case 2	25	Right salpingectomy due to right tubal pregnancy	Generalized abdominal pain of 6 hours duration of acute onset and dizziness and second week of amenorrhea	>10000	An empty uterine cavity, free abdominal fluid and coagulum in the cul-de-sac	Laparotomy (Cornual resection, followed by suturing of uterine cornu)	Successful
Case 3	39	None	Vaginal bleeding, left-lower quadrant pain and 17 days of amenorrhea	9277	Yolk sac and foetal pole within a gestational sac in the left uterine horn	Hysteroscopy followed by vacuum aspiration	Successful Negative ß-hCG levels at 25 days after the procedure
Case 4	28	None	Amenorrhea for seven weeks and lower abdominal pain	NA	Yolk sac and foetal pole within a gestational sac in the left uterine horn. CRL was measured 31 mm and foetal cardiac motion was detected A 4-cm subchorionic haematoma near the gestational sac	Vacuum aspiration under laparoscopy	Successful Negative ß-hCG levels at 7 days after the procedure

tvUS:Transvaginal ultrasound. MTX:Methotrexate. NA:Not available.

Although interstitial pregnancy is a rare condition, physicians should have a high index of suspicion, especially when there are risk factors present. In our second case, the patient’s story of laparoscopic salpingectomy due to ectopic pregnancy prompted taking this diagnosis into consideration, as ipsilateral salpingectomy has been reported as a unique risk factor for this rare type of ectopic pregnancy.^3^ No risk factors for ectopic pregnancy were seen in the first, third or fourth cases.

Diagnosis of interstitial pregnancy is generally based on ultrasonographic findings that are specific to this clinical entity, such as eccentric gestational sac, an echogenic line lying between the gestational sac and endometrium (“interstitial line sign”), a thin myometrial layer that covers the superolateral portion of the gestational sac and an empty endometrial cavity.^4^ At this point, angular pregnancy should be taken into account as a differential diagnosis. Distinction between interstitial and angular pregnancies is crucial considering that interstitial pregnancy evolves to rupture, with catastrophic outcomes, and, contrarily, angular pregnancies can progress to term. Paying attention to myometrial thickness around the gestational sac may be helpful in this matter. The myometrium is expected to be thicker at all sides of the sac in an angular pregnancy; however, myometrium less than 5 mm surrounds the interstitial gestational sac. Magnetic resonance imaging also proved to be helpful in these cases.^6^ No myometrium was seen on ultrasound images in Case 1 and 2 (Fig.1 and 2). In addition, myometrium in the superolateral portion of the sac was thinner than 5 mm in Case 4 (Fig.4). Because Case two had a presumed diagnosis of ruptured ectopic pregnancy after examination, surgery was the only option, and ultrasound meant nothing in terms of location of the ectopic gestational sac.

The most suitable management option should be considered based on individual patient factors. Conservative management in haemodynamically stable patients with falling ß-hCG was reported with a high risk of failure.^1^ The only patient in our case series to whom conservative management might have been offered was Case 1, because of low ß-hCG levels. However, no myometrium was seen on the US around the sac, and high risk of uterine rupture was considered. Therefore, conservative management was not recommended to any patient. Medical treatment such as local or systemic injection of MTX or etoposide has been reported with success rates between 66% and 100%.^7^,^8^ Early gestation, and diameter less than 4 mm and serum ß-hCG levels less than 10.000 IU/L are essential to select medical treatment in an interstitial pregnancy. Among our patients, MTX was not considered as a first option in Case 3 due to high ß-hCG levels, or in Case 4 due to presence of foetal cardiac motion. We considered systemic MTX treatment in our first case, since the inclusion criteria were all met, and the patient had a desire of future fertility. The effective decrease in serum ß-hCG between the 4^th^ and 7^th^ day after the treatment showed the efficacy of the method.

The traditional treatment for interstitial pregnancy consisted of cornual wedge resection via laparotomy.^1^ Nowadays, this option is used rarely due to development visualization tools and the wide use of serum rapid quantitative ß-hCG assays. In some selected cases, though, the cornual wedge resection remains the best choice to handle the bleeding, especially in haemodynamically non-stable patients. We had to perform this operative technique in our second case, since the patient presented haemodynamically non-stable (Fig.2). In some selected cases when conservative treatment cannot be applied, transcervical evacuation of the products of conception via hysteroscopy or under the laparoscopy guide can be considered.^5^ We performed cervical dilatation and suction of the gestational sac after hysteroscopic visualization of the gestational sac near the tubal ostium in our third case. Risk of uterine rupture, hemorrhage and need for immediate laparoscopy or laparotomy were discussed with patient. Evacuation of the whole material of conception under transabdominal ultrasound guide made the option sufficient for the management of the case. All possible surgical management options were discussed with Case 4. Laparoscopic exploration was selected first for this patient. The direct visualization of the site of an ectopic pregnancy led to successful vacuum evacuation of the conception material. Laparoscopy guidance was helpful for avoiding uterine rupture. Sufficient evacuation was proven under transabdominal ultrasound. Frozen histopathologic confirmation of the evacuated material finalized the procedure. Considering the young patient’s desire for future fertility, the selected option seems to have been the most suitable. If evacuation had been unsuccessful, laparoscopic cornual resection would have been performed. Successful laparoscopic excision with double purse-string suture technique was reported in several cases.^9^,^10^

There is no consensus on the best surgical modality for interstitial pregnancy.^5^ Minimal invasive surgeries such as laparoscopic excision, hysteroscopic excision, suction dilation and curettage under guidance of laparoscopy, hysteroscopy and/or transabdominal ultrasound are being used more commonly. From our point of view, these techniques can be performed by experienced surgeons in hemodynamically stable patients with no evidence of uterine rupture. Furthermore, patients should be counseled about the risks of uterine rupture, acut hemorrhage and possible need for laparotomy and hysterectomy.

In conclusion, our case series supported the evidence of other studies and showed that invasive management options may not be the first line treatment of unruptured interstitial pregnancies that are haemodynamically stable. Systemic MTX treatment with the same dose that is used in other types of tubal ectopic pregnancies may be an effective treatment option for those cases. Nevertheless, a radical surgical operation such as cornual wedge resection seems to be the safest approach in ruptured cases when the patient is haemodynamically unstable. Laparoscopic guided transcervical evacuation and hysteroscopic approach for the precise visualization of the site of an ectopic gestational sac can also be applied in selected cases. Prospective studies are warranted to clarify the role of non- or less invasive methods in treatment of interstitial pregnancies.

### Authors’ Contribution

**IK:** Primary surgeon of the 1st, 3rd and 4th case, literature search, analysis, writing manuscript.

**ZM:** Assistant surgeon of the 3rd case, data collecting, writing manuscript.

**HT:** Critical review.

**AU:** Data collecting, assistant surgeon in the 2nd case.

**AT:** Primary surgeon of the 2nd case, critical review.
